# Understanding multimorbidity: insights with graphical models

**DOI:** 10.1186/s12874-025-02536-y

**Published:** 2025-04-01

**Authors:** Erika Banzato, Alberto Roverato, Alessandra Buja, Giovanna Boccuzzo

**Affiliations:** 1 Department of Statistical Sciences, University of Padova, via C. Battisti 241, 35121 Padova, Italy; 2 Department of Cardiologic, Vascular, and Thoracic Sciences and Public Health, University of Padova, via L. Loredan 18, 35131 Padova, Italy

**Keywords:** Multimorbidity, Graphical model, Marginal and conditional associations, Conditional independence, Odds ratio

## Abstract

**Background:**

The use of graphical models in the multimorbidity context is increasing in popularity due to their intuitive visualization of the results. A comprehensive understanding of the model itself is essential for its effective utilization and optimal application. This article is a practical guide on the use of graphical models to better understand multimorbidity. It provides a tutorial with a focus on the interpretation of the model structure and of the parameter values. In this study, we analyze data related to a cohort of 214,401 individuals, who were assisted by the Local Health Unit of the province of Padova (north-eastern Italy), collecting information from hospital discharge forms.

**Methods:**

We explain some fundamental concepts, with special attention to the difference between marginal and conditional associations. We emphasize the importance of considering multimorbidity as a network, where the variables involved are part of an interconnected system of interactions, to correct for spurious effects in the analysis. We show how to analyze the network structure learned from the data by introducing and explaining some centrality measures. Finally, we compare the model obtained by adjusting for population characteristics with the results of a stratified analysis.

**Results:**

Using examples from the estimated model, we demonstrate the key differences between marginal and conditional associations. Specifically, we show that, marginally, all variables appear associated, while this is not the case when considering conditional associations, where many variables appear to be conditionally independent given the others. We present the results from the analysis of centrality indices, revealing that cardiovascular diseases occupy a central position in the network, unlike more peripheral conditions such as sensory organ diseases. Finally, we illustrate the differences between networks estimated in subpopulations, highlighting how disease associations vary across different groups.

**Conclusion:**

Graphical models are a versatile tool for analyzing multimorbidity, offering insights into disease associations while controlling for the effects of other variables. This paper provides an overview of graphical models without focusing on detailed methodology, highlighting their utility in understanding network structures and potential subgroup differences, such as gender-related variations in multimorbidity patterns.

## Background

The aging population is a growing concern worldwide, determining an increasing prevalence of multimorbidity, which refers to the coexistence of multiple chronic conditions within an individual [1, 2]. This phenomenon poses significant challenges for healthcare systems, caregivers, and society. Multimorbidity represents also an important research issue for understanding the associations and interactions among diseases. When analyzing multimorbidity, it is advantageous to conceptualize diseases as components of an interconnected system, where each disease interacts with the others. Representing these interactions as a network eases a more accurate estimation of the phenomenon and shifts the focus from individual disease associations with other variables (comorbidity) to the overall mechanism of interactions (multimorbidity).

Graphical modeling is a form of multivariate analysis that uses graphs to represent statistical models. It has been recognized as a valuable tool for investigating complex systems [3] and estimating causal effects from observational data [4], with successful application in fields such as genetics [5, 6] and neuroscience [7, 8], among others. These models are also gaining popularity in multimorbidity analysis due to their intuitive graphical representation of disease connections through conditional independence relationships [9, 10]. In the context of multimorbidity, it is crucial to adjust for the effect that each single variable has on all others. This adjustment is fundamental to accurately estimating and isolating the *net* associations between each pair of variables, avoiding spurious results. This is also what distinguishes this approach from other network analyses, which describe the pairwise marginal co-occurrence of diseases [11–13]. We deem that a deeper understanding of the potential of these models in the multimorbidity framework is necessary for their effective use within the epidemiological community. Therefore, we present a detailed and intuitive explanation of conditional associations as opposed to marginal associations, emphasizing the magnitude of spurious associations that might arise without adjusting for network interactions. We provide a comprehensive guide to the use of undirected graphical models, focusing on their interpretative power and understanding of the parameters involved, within a multimorbidity framework. Finally, we describe the distinction between an analysis that adjusts for a specific stratification factor and a stratified analysis, highlighting the advantages of the latter in the context of multimorbidity analysis.

### Data

**Fig. 1:**
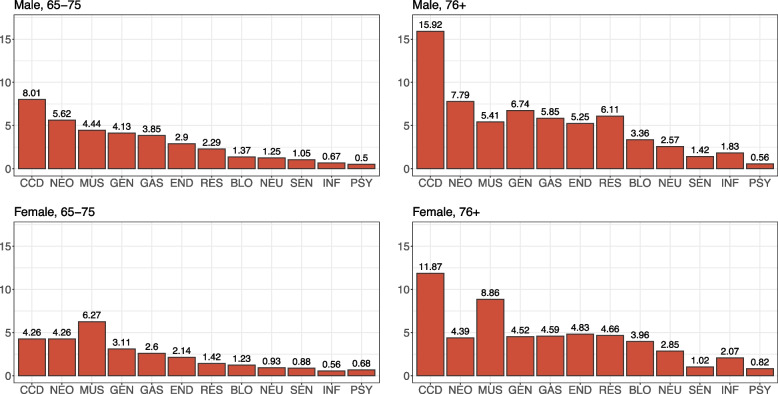
Prevalence (%) of the diseases in the subpopulations identified by the variables sex and age. For each disease, prevalence refers to events recorded if the person has at least one documented case during the 2016–2017 period. Abbreviations and IC9-CM codes of the major groups of diseases considered. PSY: psychiatric (291 - 319), BLO: blood (280 - 289), CCD: cardio-circulatory (390 - 459), NEU: neurological (320 - 359, 290, 294.1), MUS: musculoskeletal (710 - 739, 800 - 829), RES: respiratory (460 - 519), GAS: gastrointestinal (520 - 579), GEN: genito-urinary (580 - 629), END: endocrine (240 - 279), INF: infections (001 - 139), NEO: neoplasms (140 - 239, V10, V580, V581, V671, V672), SEN: sensory (360 - 389)

In this study, we analyze data related to a cohort of 214,401 individuals, who were assisted by the Local Health Unit (LHU) “ULSS6 Euganea” of the province of Padova (north-eastern Italy), and resident in the area since at least January 1, 2016. We focus the attention on the population that was 65 years or older on January 1, 2018. From hospital discharge forms, we collected information about the primary diagnosis and up to five concomitant diseases (recorded according to IC9-CM). We considered a total of 12 dichotomous variables, corresponding to 12 major category groups of diseases; see Fig. 1 for a full list. A category was considered present if the patient had at least one documented case during the 2016–2017 period.

## Methods

### Preliminaries on graphical models and graphs

In multivariate statistical analyses, which involve multiple variables, a key aspect is understanding how these variables are interdependent. A graphical model encodes such dependence structure by representing the variables as the nodes of a graph and the relationships between pairs of variables as edges. These models were first introduced in the seminal work [14], where it is explained how an undirected graph can be used to represent the conditional independence structure of a set of categorical variables. Since then, the theory of graphical models has grown to include a large number of different classes of models. The vertices of the graph can now be used to represent different types of random objects, including both quantitative and categorical random variables (see chapters 8 and 9 of [15] and [16]), as well as stochastic processes and random functions [8, 17]. A common feature of all graphical models is that the absence of an edge represents an independence relationship between the random features encoded by the corresponding nodes. However, such independence may have different forms, depending on the type of edges that are used. For instance, in undirected graphs, a missing edge represents conditional independence given all remaining variables, whereas in graphs with bidirected edges, it indicates marginal independence. The family of directed acyclic graph (DAG) models, also known as Bayesian networks [18], is extremely popular in applications and is characterized by directed edges. Although, in DAG models the interpretation of the conditional independence relationships is less straightforward than in undirected and bidirected graphs, these models naturally suit regression frameworks, as directed edges point from predictors to response variables. For this reason their use has also proved useful in the field of causal inference [19]. It is also worth mentioning that graphs are not restricted to contain a single type of edge. Accordingly, there exist classes of graphical models characterized by mixed graphs, such as chain graph models [20] and acyclic directed mixed graph models (chapter 2 of [15]), thereby allowing one to represent a wide range of different independence structures, suited for a variety of applied contexts. We close this short overview by mentioning that there is a large literature on the selection of the graph structure from data [21] and, in the last two decades, attention has shifted to high-dimensional settings where the number of variables is comparable to or larger than the sample size. This scenario arises, for instance, in microarray experiments [5]. The analysis of high-dimensional data is challenging but, fortunately, high-dimensional problems may remain tractable in the presence of structural constraints such as sparsity, that is, each node is connected to a small number of other nodes, and thus graphical models are a primary tool also in this framework [22, 23].

We now turn to the application considered in this paper, where the relevant class of graphical models consists of undirected models for binary data. Formally, an undirected graph is a mathematical object consisting of a set of nodes, $V=\{1,2,\dots ,p\}$, represented by circles, that identify the *p* variables object of study, and a set of edges, $E\subseteq \{(i,j): i \ne j,\,\, i,j\in V\}$, which are depicted as lines connecting the two corresponding nodes, i.e. variables [15, 18, 24]. Hence, if two nodes that identify two variables, say $X_i$ and $X_j$, are connected by an edge (*i*, *j*), this means that the two variables are regarded as dependent conditionally on the other variables, and a coefficient may be associated with such edge to quantify the strength of the net pairwise association between the two variables. Note that in the network, two variables can be connected directly or indirectly. An edge (*i*, *j*) encodes a direct connection between variables $X_i$ and $X_j$, whereas an indirect connection is represented by an ordered sequence of nodes and edges, called a path, joining $X_i$ and $X_j$ through other intermediate variables. In undirected graphical models, the association structure of the graph can be read as follows. When two variables are connected directly, this means that they are considered significantly associated by the model, net of the effects of all the other variables. If they are not directly connected, they are regarded as independent conditionally on the other variables, even though they might not be marginally independent. In other words, when two variables are connected directly, this means that they have an effect on each other that goes beyond the effect originating from the others. On the other hand, we may observe that two variables are conditionally independent but marginally associated when considered individually; however, this marginal association is spurious. In this scenario, there is no direct edge between the two variables, and the nodes along the path connecting these variables identify the sources of the spurious association [25].

### The model

Statistical inference for graphical models for categorical data can be carried out using log-linear models, which allow the joint estimation of all the relevant conditional odds ratios (ORs) [16, 26]. Inference for these models requires estimating parameters for the main effects of the variables $(\lambda _i, i=1,\dots ,p)$, parameters for the interactions between pairs of variables $(\lambda _{ij}, i\ne j, i=1,\dots ,p)$, but also all the possible parameters for the three-way and higher-order interactions. However, it is important to note that when the number of variables is large, the contingency table is typically sparse, in the sense that many of its cells are empty, even when the sample size is large. This is the case of our multimorbidity dataset that, despite the very large sample size, produces a contingency table where only $32.7\%$ of cells are non-empty. In sparse tables, estimating the parameters of log-linear models is challenging. A common solution to this problem is to consider a subfamily of graphical models known as the Ising model [27], which involves only main effects and pairwise interactions. This approach quantifies the strength of the conditional association between two variables by means of a single odds ratio that determines the presence or absence of the corresponding edge in the graph. Formally, the Ising model consists of one main effect, $\lambda _{i}$, for each variable $X_i, i=1,\dots ,p$, and one pairwise interaction $\lambda _{ij}$ for each pair of variables $X_i$ and $X_j, i\ne j, i,j=1,\dots ,p$. Each pairwise interaction, $\lambda _{ij}$, is associated with an edge of the graph, (*i*, *j*), that is not present if $\lambda _{ij}=0$. It follows that statistical inference involves learning the structure of the graph by identifying the pairwise interactions that are not significantly different from zero, and then estimating the model parameters to quantify the strength of the associations. It is worth remarking that to learn the graph structure, it is sufficient to identify which edges can be removed from the graph, meaning which pairwise interactions in the Ising model are equal to zero. When the number of variables is large, this structure learning step can be conveniently carried out locally by considering one variable at a time. The edges connecting each variable to the remaining variables are then identified using a regression model. For binary variables, this involves estimating a logistic regression for each variable, against the remaining ones [23, 27, 28].

### Model estimation

We implement model estimation in two steps: the first step involves structure learning, while the second step involves estimating the model parameters. To learn the structure, we estimate node-wise logistic regressions for each variable against the others [29]. For each logistic regression, the relevant explanatory variables are identified by a stepwise, backward elimination, selection method based on the Extended Bayesian Information Criteria (EBIC), that is a generalization of the classical BIC [30], specifically designed to deal with the graphical model framework [28, 29, 31]. The (local) structure of the graph is then obtained by connecting with an edge the response variable with each of the remaining variables resulting from the model selection procedure. Next, we estimate the Ising model corresponding to the structure learned in the initial step. This is done by maximum likelihood estimation, thereby jointly estimating the main effects and the pairwise interaction parameters corresponding to the edges of the structure identified in the previous step. The model was identified by also including the variables sex and age (65–75, 76+), which can be regarded as additional nodes in the graph.

### Centrality measures

After estimating the network, we can proceed to analyze its structure. This type of analysis can be performed by computing measures specifically designed to quantify the relevance of a node according to different definitions of centrality [32, 33]. In this study, we focus on two indices: the closeness and the betweenness centrality (Table 1), which are especially appealing from an interpretative standpoint. The closeness of a node is defined as the inverse of the sum of the distances between that node and all other nodes in the graph, calculated via the shortest paths. Therefore, the more central a node is, the closer it is to all other nodes in the network. Betweenness is a measure of centrality for vertices within a graph and evaluates how frequently a node serves as a bridge along the shortest path connecting two other nodes. For both measures, closeness and betweenness, higher values indicate a more central position of the node within the network.

**Table 1:** Prevalence (%) and normalized measures of closeness and betweenness centrality. Variables are ordered according to their closeness values. For each disease, prevalence refers to events recorded if the person has at least one documented case during the 2016–2017 period

Major disease group	Prevalence ($\%$)	Closeness	Betweenness
Cardio-circulatory (CCD)	9.66	1	1
Blood (BLO)	2.48	0.520	0.185
Neurological (NEU)	1.89	0.460	0.111
Respiratory (RES)	3.48	0.434	0
Psychiatric (PSY)	0.66	0.432	0.333
Endocrine (END)	3.72	0.419	0
Gastrointestinal (GAS)	4.11	0.377	0.148
Infectious (INF)	1.28	0.327	0
Genitourinary (GUR)	4.47	0.312	0.111
Musculoskeletal (MUS)	6.43	0.256	0.037
Neoplasm (NEO)	5.31	0.095	0.037
Sensory (SEN)	1.07	0	0

### Subgroup analysis

When analyzing data with graphical models, it is important to consider that networks can vary between subgroups of the population, due to their specific characteristics, such as sex or age. In the model described in the previous section, we opted to add two nodes, one for each variable. A strategy like this amounts to assuming that the structure remains consistent across all groups, while acknowledging potential differences in disease occurrence based on sex and age. The output of the estimation procedure yields potential edges connecting the variables sex and age with the diseases, while edges connecting two diseases are interpreted as conditional associations net of the effect of sex and age. However, the coefficient associated with the edge is the same regardless of sex and age. Alternatively, separate analyses for each subgroup can be performed, considering sex and age as stratification variables and allowing the structures within the groups to vary. This approach acknowledges the potential heterogeneity of the graph structure itself within the population and enables a more comprehensive understanding of the networks, also in terms of the strength of the connections. Considering the demographic tendency where the women population generally has a higher average age compared to the men population, we recommend balancing the two samples by age. In this application, we extracted an equivalent number of women and men within each five-year age group to address any imbalance that might lead to misleading results.

## Results

The prevalence of the main categories of diseases are shown in Table 1. Prevalence refers to the years 2016–2017 and is calculated as the total number of events during this period divided by the individuals aged 65 years and older. For each disease, an event is recorded if the person has at least one documented case during the 2016–2017 period. Note that the most prevalent condition is cardio-circulatory disease, $9.66\%$, while the least common is psychiatric disease, $0.66\%$. Figure 1 provides the prevalence of the diseases in the subpopulations identified by sex and age. The prevalence rates vary across the groups. In particular, the prevalence is higher among individuals aged 76 and older, while men tend to have a higher prevalence of most diseases compared to women. However, musculoskeletal diseases are an exception, with a higher prevalence observed in the female group.

### From marginal to conditional independence

**Fig. 2:**
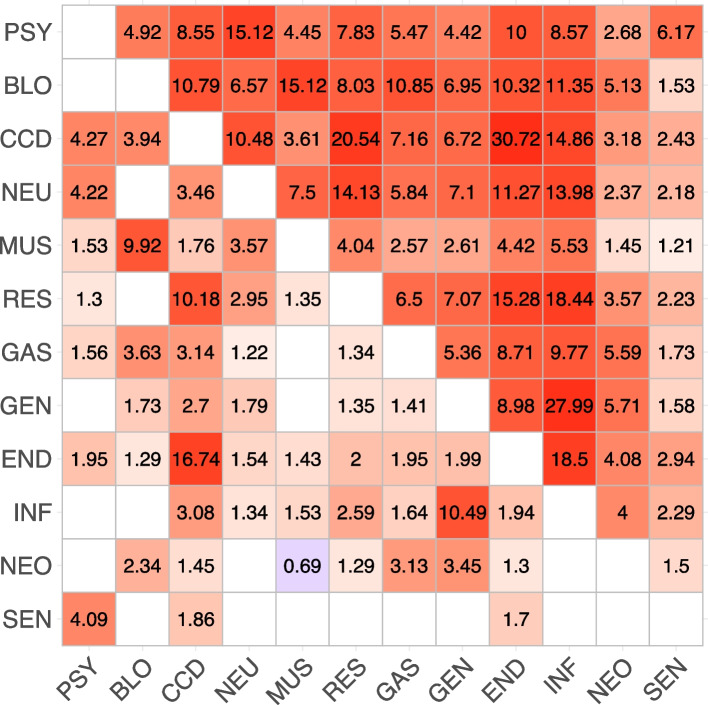
Matrix of marginal and conditional odds ratios. The conditional ORs are reported in the lower triangular part of the matrix, while the marginal ORs are in the upper triangular part. The conditional OR are adjusted for sex and age (65–75, 76+). Blank cells indicate conditional independence of the relative variables in the estimated model. The color of the cells is proportional to the Yule’s *Q* coefficient computed using the  estimated OR: red cells represent positive associations, while blue cells stand for negative associations

**Fig. 3:**
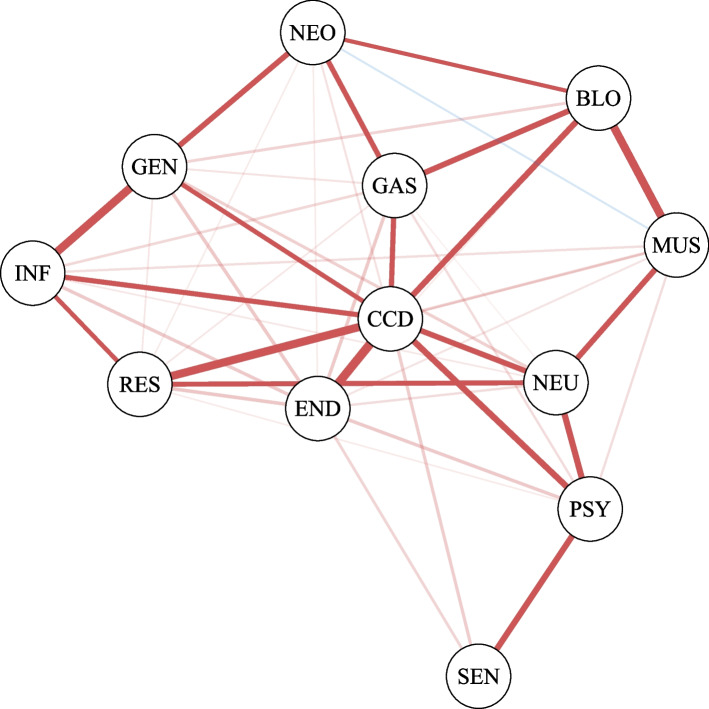
Estimated network for the entire population aged 65 and over. Blue lines represent negative associations, while red lines stand for positive associations. The thickness and the color of the lines are proportional to the Yule’s *Q* coefficients calculated on the estimated ORs. Edges with no transparency have a $Q>0.4$ and correspond to the strongest associations. Each edge is associated to an odds ratio reported in the lower triangular part of the matrix in Fig. 2. Nodes relative to the sex and age variables were omitted to emphasize the structure of the multimorbidity network

The word *conditionally*, as opposed to *marginally*, is of primary importance for the interpretation of the association structure encoded by an independence graph. The marginal odds ratio provides information on the marginal associations between each pair of variables, whereas the conditional odds ratio provides information related to conditional associations, which are the net pairwise associations after accounting for the influence of other variables. The upper triangular part of the table in Fig. 2 shows the pairwise marginal odds ratios, computed from the multimorbidity data. The number of marginal odds ratios to be computed and assessed for statistical significance is given by $p \, \times (p-1)/2$. In our case, this results in 66 tests. Conducting such a large number of tests places our analysis within the context of multiple testing. Given that we perform 66 tests at a significance level of $\alpha =0.05$, the probability that at least one rejected hypothesis is a false positive is much higher than the nominal significance level $\alpha$. This probability is called the family wise error rate (FWER). In this context, it is crucial to control it, ensuring that the probability of making at least one type I error across the family of tests remains within acceptable limits. To address this issue, various methods exist for controlling the FWER [34]. In particular, we use the Holm method [35], which is suitable regardless of the dependence structure of the *p*-values. Specifically, after applying the Holm correction, all odds ratios in Fig. 2 remain significantly greater than 1. This finding suggests a robust positive association between each pair of variables, even after accounting for multiple testing. It follows that the analysis of the marginal odds ratios does not reveal any non-obvious association structure, suggesting that all disease categories are associated with each other. However, marginal pairwise associations might be influenced by the effects of the other variables in the system. In the remainder of this section, we show how the odds ratios may change when computed conditionally on these additional variables. Consider, for example, the two variables related to sensory diseases (SEN) and genitourinary diseases (GEN). The odds ratio calculated on the marginal $2\times 2$ table shows a positive and significant association (OR=1.58, *p*-value $<0.001$); see Fig. 2. However, the interpretation of this association is not straightforward in clinical terms. On the other hand, one can consider the conditional odds ratio, that is the odds ratio computed on the $2\times 2$ table for some fixed levels of the other variables. More specifically, there exist $2^{10}\ 2\times 2$ tables of sensory and genitourinary diseases, one for each of the possible level combinations of the remaining 10 variables. Hence, $2^{10}$ conditional odds ratios can be computed accordingly, and two variables are said to be conditionally independent of each other given all the remaining variables if all the conditional odds ratios are equal to 1. We remark that the conditional independence structure of a set of variables can be effectively visualized using a graph, alternatively referred to as a network. Figure 3 shows the graph that we learned from the multimorbidity data. To focus on the multimorbidity network, we chose to display only the nodes corresponding to the major disease groups, omitting those related to sex and age from the visualization. We now return to the example of genitourinary diseases and sensory diseases. As discussed previously, the two variables are significantly marginally associated. However, the graph in Fig. 3 shows that the corresponding nodes, SEN and GEN, are not directly connected; instead, they are indirectly connected through a series of paths, e.g. SEN-PSY-CCD-GEN.

### Interpretation of model parameters

The parameterization of the Ising model has an interpretative advantage, namely the conditional independence relationship between two variables, say $X_i$ and $X_j$ is represented by a single odds ratio, that is given by $e^{\lambda _{ij}}$. Hence, $\lambda _{ij}$ is the logarithm of the conditional odds ratio so that if $\lambda _{ij}=0$, then $X_i$ and $X_j$ are conditionally independent given the other variables. The interaction values we learned from the multimorbidity data are shown in Fig. 2, where the lower triangular part of the matrix gives the conditional odds ratios jointly estimated by the Ising model. The empty cells represent the missing edges in the graph shown in Fig. 3, which correspond to estimated conditional odds ratios not significantly different from 1. By estimating the model, we can now identify a structure that we could not see with the marginal odds ratios in the upper triangular part of the matrix in Fig. 2. Odds ratios not significantly different from 1 imply lack of associations, positive values significantly smaller than 1 represent negative associations, whereas values significantly greater than 1 represent positive associations. It follows that the odds ratio is an asymmetric measure of association and, for this reason, it is not suited to be used in the graphical representation. Instead, we transform the odds ratios into the Yule’s *Q* coefficients. The Yule’s *Q* is computed as (OR-1)/(OR+1), and its values range between $-1$ and 1, with a value of zero indicating no association [36]. In Fig. 3, the red color is used for positive associations, whereas the negative associations are given in blue. The thickness and intensity of the edges color are proportional to the strength of the corresponding associations, measured by the Yule’s *Q*.

### Topological analysis of the graph

After estimating the network, it becomes interesting to analyze the topology of the graph to infer the importance of the variables within the system. First, we calculate the density of the graph, which is a proportion between 0 and 1, calculated as the number of edges of the graph divided by the total possible number of connections, $p(p-1)/2$. The graph in Fig. 3 has density $69.7\%$. Despite the dense graph, we can observe that the strength of associations for many edges is weak, with only a limited number of edges exhibiting substantial relevance. Particularly notable is the position of the cardio-circulatory system diseases (CCD) node, which acts as a hub, being directly connected with strong association values to almost all other nodes, thereby highlighting the central role played by this variable. This also suggests that this variable may play an important role in determining the strong spurious associations that we observed in the marginal odds ratios. On the other hand, we can also note that in the network, the node representing sensory diseases (SEN) is peripheral, indicating its position on the boundary rather than at the core of the network.

Table 1 shows the values of closeness and betweenness centrality. Values are normalized, ranging between 0 and 1, where 1 indicates the most central node and 0 the most peripheral. As stated previously, visual inspection of the graph in Fig. 3 reveals that cardio-circulatory diseases (CCD in the graph) appear to play a central role in the system. This is also confirmed from the values in Table 1, which state that the node of cardio-circulatory diseases is the most central one, as it has both high closeness and betweenness indices. On the other hand, the node relative to sensory diseases (SEN in the graph in Fig. 3) is the more peripheral one because it shows low values of both indices.

### From the global population network to the subpopulations networks

**Fig. 4:**
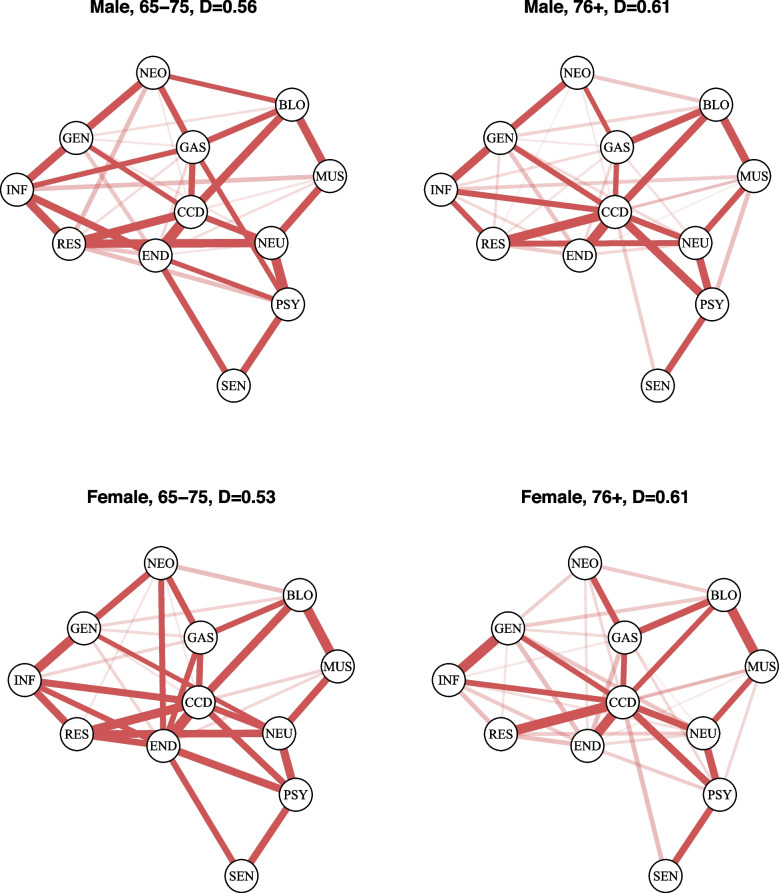
Estimated graph in four subpopulations defined by age and sex. D is the density index. All the conditional associations are positive and represented by red lines. The thickness and the color of the lines are proportional to the Yule’s *Q* coefficients calculated on the estimated ORs. Edges with no transparency have a $Q>0.4$ and correspond to the strongest associations

Figure 4 gives the estimated graphs in the four subgroups (men 65–75 years old, women 65–75 years old, men 76 and older, and women 76 and older), the position of the nodes was kept fixed to ease the comparison between the groups. Looking at the four networks in Fig. 4, one can note that the densities of the networks are diminished compared to the density of the global population network in Fig. 3. For both the female and male groups, the networks of the $76+$ populations exhibit a higher density compared to the networks of the younger subjects. Moreover we can also note that for the 65–75 group, the male graph is denser. However, it is interesting to note that, in all subgroups, the cardio-circulatory system diseases (CCD) group remains a central node; as a matter of fact, cardiovascular diseases are known to be involved in different clinical pathways that link different system diseases, in both female and male sexes.

**Fig. 5:**
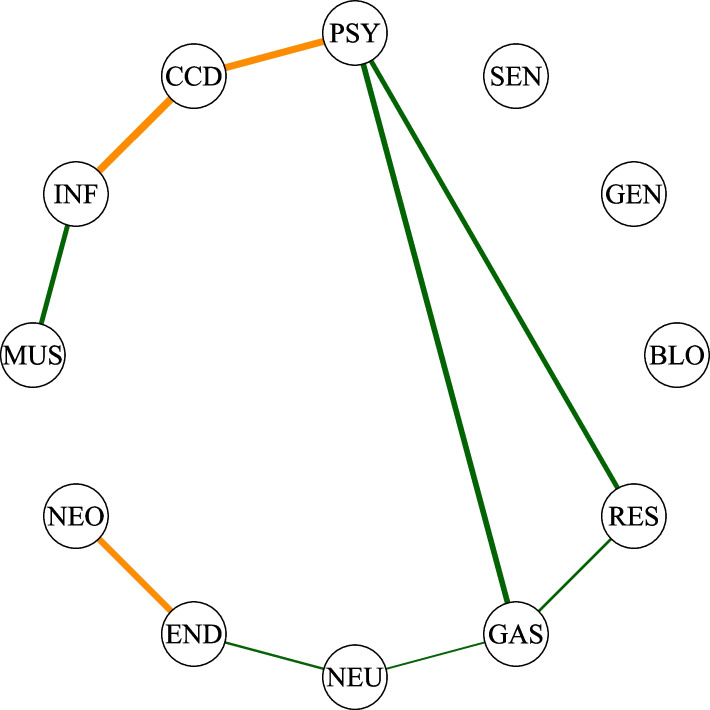
Differences between male and female networks, for the 65–75 age group. Green and orange lines represent associations present in one group but absent in the other, for men and women respectively. The thickness of the lines corresponds to the strength of the associations and is proportional to the Yule’s *Q* computed on the conditional ORs estimated by the subpopulation models

Differences in densities imply differences in the number of conditional independence relationships, suggesting that some associations exist in one population but not in another. An example can be found in Fig. 5, where, for the 65–75 age groups, we show the differences between male and female networks. Specifically, the green and orange lines represent associations present in one group but absent in the other, for men and women, respectively. The main advantage of conducting a stratified analysis is that the relationship between two variables can be estimated separately within each stratum. This approach helps to understand whether the association between the main variables differs between different subgroups. Consider, for example, the association shown in Fig. 3 between neoplasms (NEO) and endocrine diseases (END) in the general population network. The conditional odds ratio of the association is 1.3 (Fig. 2) and represents the effect that the variables have on each other, net of the effect of the other groups of diseases, sex and age. If we look again at the association between neoplasms (NEO) and endocrine diseases (END) in Figs. 4 and 5, we observe that in the subgroup of male individuals aged 65–75, the edge is missing, indicating that the two variables are conditionally independent given the other groups of diseases. On the other hand, if we look at the subgroup of women aged 65–75 years, we see the presence of the edge suggesting the existence of an association with an odds ratio equal to 3.14.

This analysis shows that when we consider a separate network for each subpopulation, then for every pair of variables, $X_i$ and $X_j$, we estimate an interaction $\lambda _{ij}$ for each subpopulation, that is equal to zero when the edge of the relevant subpopulation network is equal to zero. It can therefore be of interest to compare such interactions or the corresponding ORs. In the example above, we compared the conditional ORs of the association between neoplasms and endocrine diseases between subpopulations in a naïve way. However, we remark that formal hypothesis testing comparison can be performed with standard inferential methods. It is also worth remarking that in the approach in which a single global population network is estimated, based on an Ising model, a single $\lambda _{ij}$ interaction is estimated for every pair of variables (Fig. 3). If the effect of sex and age on such interactions is of interest, then this can be assessed by extending the Ising model to include higher-order interactions. It is also important to recall that when a large number of comparison is of interests, then suitable corrections for multiple tests should be applied.

## Discussion

Understanding the conditional associations among comorbidities is crucial for effective patient care, treatment planning, and health outcome optimization. Conditional associations go beyond mere coincidence, often reflecting shared risk factors, biological pathways, or causal relationships [37].

In this study, we observed that the densities of the networks in the younger population were lower compared to the density of the networks in the older population. These findings suggest that the increased density in the networks of older individuals may reflect differences in interaction patterns and health dynamics across age groups. This could be interpreted as a consequence of the fact that, with the course of life, a primary disease can influence the development or exacerbation of secondary conditions. However, among the youngest groups, we note that the female graph displays a lower density compared to the male graph. These findings may be explained by the fact that the onset of many conditions can be hormonal or behavioral (e.g., smoke); both factors are differently distributed in sex. Consequently, for example, estrogens have a cardiovascular protective effect in women during the premenopausal period and may have a more frequent later onset in cardiovascular diseases [38]. In contrast, in the 65–75-year-old male group, it appears to find a more advanced progression of pathological pathways and an advanced interaction with other conditions compared to women [39].

Stratified analysis also revealed notable differences in the association between diseases, such as neoplasms (NEO) and endocrine diseases (END), across subgroups. We observed that in the male subgroup aged 65–75, the two variables were conditionally independent, whereas in the corresponding female subgroup, a significant association was present. This finding is consistent with existing evidence in the literature. It is widely known that diabetes is associated with an increased risk of cancer in both sexes, but a wide meta-analysis found that the maximum-available-adjusted pooled sex-specific relative risk (RR) estimates for combined fatal and non-fatal cancer associated with diabetes were 1.27 ($$95\%$$ confidence interval (CI): 1.21, 1.32, *p*-value$$< 0.001$$) for women and 1.19 ($$95\%$$ CI: 1.13, 1.25, *p*-value $$< 0.001)$$ for men. The pooled women-men ratio of RRs was 1.06 ($$95\%$$ CI: 1.03, 1.09, *p*-value$$< 0.001$$) [40]. Moreover, neoplasia of hormone-responsive tissues currently accounts for almost 50% of all newly diagnosed cancers in females, compared to nearly 35% in males in the United States. In women, in addition to endogenous hormones that could affect the risk of these cancers and their overall frequency, there are also hazards associated with hormones administered for therapeutic purposes (e.g., as contraceptives, hormone replacement therapy, or for the prevention of miscarriage) [41]. Another interesting finding is that the connection between cardiovascular diseases (CCD) and psychiatric conditions (PSY) is observed in women but not in men in the 65–75 age group, as previously reported in the literature. In fact, studies demonstrated that women’s brains are more sensitive to factors affecting mental health, such as depression and stress, than men’s brains. In women, poor mental health increases the risk of cardiovascular disease, and conversely, cardiovascular disease increases the incidence of mental illness such as depression. In connection with mental health and cardiovascular health, the presence of gender differences in brain activation, cortisol secretion, autonomic nervous system, vascular health and inflammatory response has been observed [42]. This connection suggests that strategies to manage women’s mental health can contribute to preventing cardiovascular disease.

Understanding multimorbidity patterns aids in early detection and more accurate risk stratification. For instance, the association between metabolic disease (e.g. diabetes) and neoplasm disease has led to enhanced screening protocols and preventive strategies. Awareness of comorbidity interactions allows for more tailored treatment approaches. For example, recognizing the high prevalence of depression in patients with cardiovascular diseases in female has led to integrated treatment models that address both conditions simultaneously [42]. Recognizing patterns of comorbidities enables healthcare systems to better allocate resources and design care pathways that address the needs of patients with specific combinations of conditions [43]. Moreover, studying conditional associations informs the development of more comprehensive clinical guidelines and directs research towards addressing gaps in managing complex health profiles [44].

Regarding gender differences, understanding the variations in multimorbidity patterns between genders is essential for effective clinical management. Biological differences between males and females, including genetic, hormonal, and physiological factors, can significantly influence disease development, progression, and treatment responses [45]. Generally, most diseases affecting both sexes, such as cancers, immune-mediated disorders, cardiovascular conditions, and infectious diseases, exhibit differences not only in frequency, clinical manifestations, progression, prognosis, symptoms, and treatment response, but also in their interactions. The study of sex-specific clinical pathways linking various comorbidities, as demonstrated in our research on subgroup differences in comorbidity associations between sexes, is not merely an academic pursuit but an essential clinical requirement for prevention and treatment strategies. However, this knowledge also enables healthcare planners to adequately prepare for the needs of an aging population and develop effective public health strategies [46]. Additionally, depicting sex-specific comorbidities contributes to health equity by addressing potential disparities in care and outcomes between men and women [47]. In general, by focusing on these gender-based differences, researchers and healthcare providers can develop more comprehensive and nuanced approaches to patient care, ultimately improving health outcomes for both sexes [48].

Therefore, as the complexity of patient health profiles increases, understanding the conditional associations among comorbidities becomes paramount. This knowledge not only improves individual patient care but also contributes to more efficient and effective healthcare systems overall.

## Conclusion

Graphical models offer a versatile approach to understanding multimorbidity, providing valuable insights into the associations between disease variables while also controlling for the effect of other variables. These tools are crucial for researchers to interpret network structures through their parameters, allowing a deeper understanding of multimorbidity patterns. The use of graphical models could also be extended to other areas of health research, for example, to study modifiable risk behaviors that cluster within individuals, which implies that these behaviors are not randomly distributed across the population but tend to occur together in certain individuals. Other possible applications include studying the association between different types of disability, both physical and mental, or perceived health (e.g. examining the relationships between items in the SF-36 quality of life survey), as well as cancer, genomics, and neuroimaging. The purpose of this paper was to provide a general overview of the potential of graphical models, without delving into detailed methodological aspects and various possible alternatives. Beyond these initial steps, researchers can further explore the network topology, using measures such as closeness and betweenness centrality to assess the importance of nodes within the network. However, other centrality measures exist as well, and the selection of which measure to use depends largely on the specific goals and objectives of the analysis. In addition, community detection methods offer tools to identify clusters of closely connected nodes, revealing potential subgroups within the variables. It is worth noting that networks may differ across subpopulations. Exploring this phenomenon could provide a deeper understanding of the complex dynamics that influences multimorbidity and allow a more targeted approach to identify relevant associations for each subgroup.

Undirected graphical models represent a flexible and powerful tool for understanding associations between variables, but it is important to clarify that they do not aim to explain causal relationships unless there are underlying assumptions about the model that go beyond the scope of the method itself. More specifically, graphical models suited to describe causal relationships typically involve also directed edges and allow for both marginal and conditional independence relationships. Such marginal independence relationships cannot be represented by an undirected graphical model that is expected to show a spurious edge instead [19]. Understanding the mechanisms underlying the associations estimated with graphical models requires further investigation based on research hypotheses and the structure of the study.

## Data Availability

The data supporting this study’s findings are held by the 6 LHU-Veneto region and were used under license for this work, but they are not available to the general public.

## Abbreviations

BIC: Bayesian information criteria
BLO: Blood diseases
CCD: Cardio-vascular diseases
CI: Confidence interval
DAG: Directed acyclic graph
EBIC: Extended bayesian information criteria
END: Endocrine diseases
GAS: Gastrointestinal diseases
GEN: Genitourinary diseases
INF: Infectious diseases
LHU: Local health unit
NEO: Neoplasms
NEU: Neurological diseases
MUS: Musculoskeletal diseases
OR: Odds ratio
PSY: Psychiatric diseases
RES: Respiratory diseases
RR: Relative risk
SEN: Sensory diseases

## Code availability

The code necessary for reproducing the analyses presented in this study is available at the following link https://github.com/ebanzato/MorbiGraph. This code, written in R language [49], is accompanied by detailed documentation to assist users in its application. Providing these resources aims to support transparency and facilitate the replication and extension of our findings.
